# Management of Unsatisfactory Postoperative Double Eyelid With Intralesional Corticosteroid Injection

**DOI:** 10.3389/fmed.2021.619547

**Published:** 2021-03-24

**Authors:** Siyi Zhang, Yixiong Zhou, Fei Yu, Dan Yan, Yan Yan, Meng Zhou, Yao Fu, Yang Lu

**Affiliations:** ^1^Department of Ophthalmology, Shanghai Ninth People's Hospital, Shanghai JiaoTong University School of Medicine, Shanghai, China; ^2^Shanghai Key Laboratory of Orbital Diseases and Ocular Oncology, Shanghai, China

**Keywords:** double eyelid, management, unsatisfactory outcomes, intralesional injection, corticosteroid injection

## Abstract

**Purpose:** The present study was designed to observe the curative effect of early intralesional corticosteroid injection to treat unsatisfactory double eyelid.

**Methods:** This retrospective, observational study included 30 female patients (age 22–35 years) receiving intralesional corticosteroid injection after complaining about the unsatisfactory double eyelid post-transcutaneous upper eyelid blepharoplasty. The postoperative double eyelid anomalies included multiple folds, sunken eyelids, and severe postoperative eyelid edema. The evaluation of the clinical effect was based on the bilateral symmetry of the eyelid, the placement of the lid fold, the radian of the double eyelid, and the extent of the swelling and depression.

**Results:** Multiple folds were observed in 11 (36.67%) patients, sunken eyelids in 9 (30.00%) patients, and severe postoperative eyelid edema in 10 (33.33%) patients. Among them, 17 (56.67%) patients received one intralesional injection and experienced significant improvement in the eyelid, while 8 (26.67%) accepted another injection at 1 month after the first injection and achieved marked improvement. The remaining 5 (16.67%) patients were dissatisfied with the results and might need further repair surgery: 2 patients had multiple folds, 2 showed postoperative edema, and 1 presented sunken eyelids. None of the patients reported any adverse reactions.

**Conclusion:** Intralesional corticosteroid injection for the correction of the postoperative unsatisfactory double eyelid is safe and convenient, and provides a novel method for early intervention.

## Introduction

Asians have a more prominent preseptal fat distribution, variable position and depth of the superior palpebral fold, and insufficient or absent terminal interdigitations of levator aponeurotic fibers onto the pretarsal skin and orbicularis compared to Caucasians (1, 2). Therefore, some Asians have a single upper eyelid, upper eyelid hypertrophy, or downward growing eyelashes, which makes their eyes appear small and droopy (2, 3). Thus, double eyelid blepharoplasty is one of the most commonly performed cosmetic surgical procedures and has become increasingly popular in Asia, especially in young women, for modifying the eyes with a pair of attractive double eyelid (4, 5).

However, with the frequent performance of double-eyelid blepharoplasty, postoperative complications, such as unsatisfactory results, dry eye disease, and lagophthalmos, are also common (6, 7). Unsatisfactory double eyelid is the most frequent complication and the primary cause of the contradiction between patients and doctors, thereby necessitating that these complications be addressed appropriately and promptly. Traditionally, the unsatisfactory double eyelid was repaired by a second operation after a minimum of 3-month interval post the first operation, and corrective methods are based on the cause of the complication (8–12). However, a second operation could cause further damage to the patient and aggravate the scar formation. In addition, the outcome of the repair surgery is uncertain and uncontrollable, and the recovery was prolonged. Also, the long wait for the second operation is torturous for patients and psychologically damaging. These major concerns require a rapid and effective intervention for the unsatisfactory postoperative double eyelid.

Injection of corticosteroid is commonly and widely used for the treatment of various musculoskeletal and dermatological conditions (13, 14). The intralesional injection of corticosteroid is one of the first-line options to treat scars (15). The unsatisfactory postoperative double eyelids are mainly due to the unrelieved edema and scar formation (8). Hence, corticosteroid injection could correct the unsatisfactory double eyelid since this nonsurgical intervention is cost-effective and convenient and avoids the second surgery. To the best of our knowledge, the outcome of early corticosteroid injection in correcting unsatisfactory double eyelid has not yet been summarized. Therefore, this retrospective study was designed to observe the curative effect of early intralesional corticosteroid injection to treat the unsatisfactory double eyelid and evaluate the morphological changes after the injection.

## Methods

This retrospective, observational study was conducted in compliance with the tenets of the Declaration of Helsinki, and approved by the ethics committee of Shanghai Ninth People's Hospital.

### Participants

A total of 30 female patients referred to the Department of Ophthalmology of Shanghai Ninth People's Hospital because of unsatisfactory postoperative double eyelid, were included in this study. Different surgeons operated on these patients. In this cohort, the postoperative double eyelid anomalies included multiple folds, sunken eyelids, and severe postoperative eyelid edema. The anomalies, such as the disappearance of the double eyelid, upper eyelid weakness, and deformation of the upper tarsal margin caused by postoperative infection, which could not be treated by corticosteroid injection, could not be included in this study. Also, patients with a history of multiple lid surgeries and postoperative inflammation were excluded from the present study.

### Intralesional Injection

**Figure 1:**
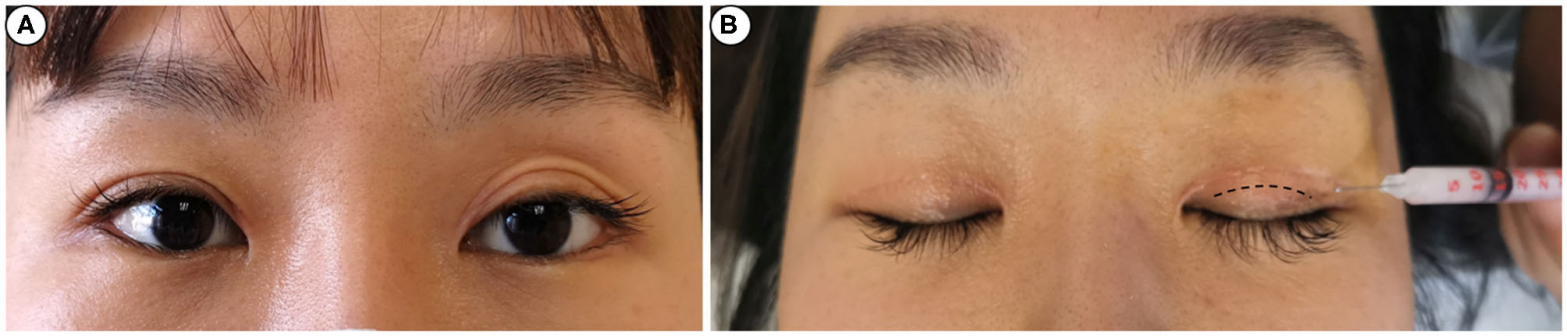
Schematic illustration of injection. A 26-year-old woman **(A)** complained of multiple folds on the left side. The needle is inserted 3-4 mm beneath the incision from the outside and injected along the dotted line (parallel to the incision) **(B)**.

All the patients signed informed consent before receiving the intralesional injection. The treatment was initiated at 3 weeks after the operation. One milliliter vials containing 7 mg of betamethasone (Diprospan®, Schering-Plow Labo N.V., Belgium) were mixed with 0.3 mL of lidocaine. A volume of 0.1–0.3 mL (containing 0.5–1.6 mg of betamethasone) was injected intradermally at a distance of 3–4 mm beneath the incision according to the severity of double eyelid anomalies (Figure 1) (Supplementary Video 1). All the injections were administered by the same surgeon (YL).

### Evaluation of the Clinical Effect

Patients were followed up at 1, 3, and 6 months after the injection. At the 1-month and 6-month follow-up appointment, each patient was subjected to a clinical assessment with respect to the morphology of the double eyelid (Table 1), which included the bilateral symmetry of the eyelid, the placement of the lid fold, the radian of the double eyelid, and the extent of the swelling and depression.

**Table 1:** Assessment of the morphology of the double eyelid after injection at different follow-up appointments.

	**1-month post-injection**			**6-month post-injection**		
	**Satisfied**	**Improved**	**Unsatisfied**	**Satisfied**	**Improved**	**Unsatisfied**
Bilateral symmetry of the eyelid	40.00%	30.00%	30.00%	63.33%	26.67%	10.00%
Placement of the lid fold	36.67%	33.33%	30.00%	56.67%	30.00%	13.33%
Radian of the double eyelid	43.33%	43.33%	13.33%	73.33%	23.33%	3.33%
Extent of the swelling	43.33%	46.67%	10.00%	76.67%	20.00%	3.33%
Extent of the depression	53.33%	36.67%	10.00%	73.33%	16.67%	10.00%

### Criteria for a Second Injection

If the clinical assessment of the morphology of the double eyelid was unsatisfactory at 1 month after the first injection, the patients would accept a second injection.

### Statistical Analysis

According to the literature review, the effective rate of the traditional surgical method is 95% (8, 9), and the effective rate of the current method was estimated to be 80%. A two-sided α 0.05 and β 0.2 were applied. A sample size of 26 cases was calculated using PASS15.0 (NCSS, Kaysville, UT, USA), and 30 cases were included in this study.

## Results

**Figure 2:**
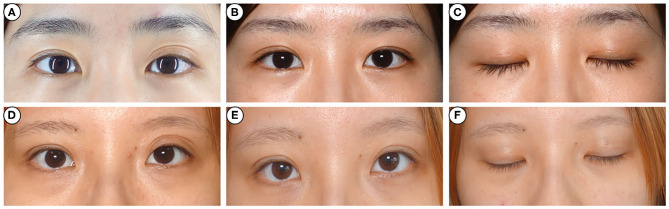
Photographs before and after injection. A 28-year-old woman **(A)** and a 24-year-old woman **(C)** complained of multiple folds on the left side 3 weeks after transcutaneous upper blepharoplasty. They were administered intralesional corticosteroid injection once, and the appearance improved markedly after 6 months **(B,D)**. And there was no skin atrophy or angiotelectasis **(E,F)**.

**Figure 3:**
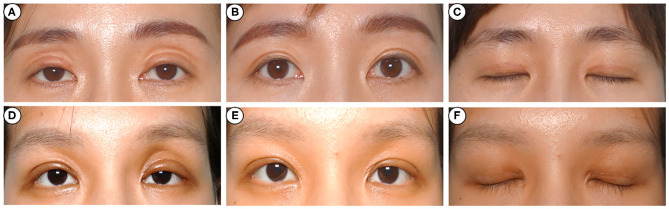
Photographs before and after injection. A 32-year-old woman **(A)** complained of sunken eyelids on both sides 22 days after transcutaneous upper blepharoplasty. She was administered intralesional corticosteroid injection twice, and the appearance improved remarkably after 6 months **(B)**. A 26-year-old woman **(C)** complained of sunken eyelids on the left side 3 weeks after transcutaneous upper blepharoplasty. She was injected with intralesional corticosteroid once and displayed significant cosmetic improvement after 6 months **(D)**. And there was no skin atrophy or angiotelectasis **(E,F)**.

**Figure 4:**
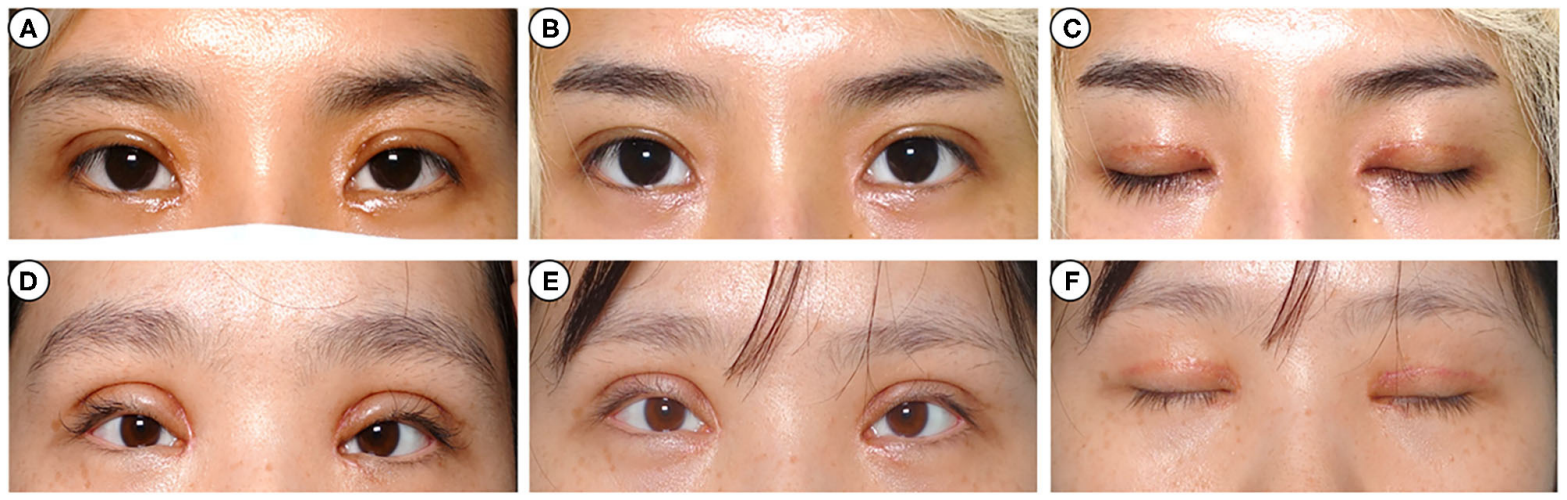
Photographs before and after injection. A 24-year-old woman **(A)** and a 28-year-old woman **(C)** complained of eyelid edema on both sides 3 weeks after transcutaneous upper blepharoplasty. They were injected once with intralesional corticosteroid, and the appearance improved significantly after 6 months **(B,D)**. And there was no skin atrophy or angiotelectasis **(E,F)**.

A total of 30 patients (41 eyes) were followed up for 6 months to observe the clinical effect after corticosteroid injection. The mean age of the participants was 29.07 ± 3.46 (range, 22–35) years. Among these patients, multiple folds were observed in 11 (36.67%) patients (Figures 2A,C), sunken eyelid in 9 (30.00%) (Figures 3A,C), and severe postoperative eyelid edema was observed in 10 (33.33%) patients (Figures 4A,C).

After 1 month post-first intralesional injection, 17 (56.67%) patients displayed significant improvement in the morphology of the double eyelid. However, the remaining 13 (43.33%) patients did not undergo obvious change and accepted a second injection. Among these 13 patients, 5 presented multiple folds, 4 showed sunken eyelid, and remaining had severe eyelid edema. After the second injection, 5 (16.67%) patients were still dissatisfied with the results and might prefer an additional corrective surgery to repair the eyelid. Among the 5 patients, 2 had multiple folds, 2 had postoperative edema, and 1 had sunken eyelid. The condition of corrective corticosteroid injection of unsatisfied double-eyelid is summarized in Table 1.

A marked cosmetic improvement was noted in 9 patients with multiple folds (Figures 2B,D), 8 with sunken eyelid (Figures 3B,D), and 8 with postoperative eyelid edema (Figures 4B,D). However, none of the patients reported any allergic reaction, increased intraocular tension, skin atrophy, angiotelectasis or other corticosteroid injection-related complications (**Figures 2E,F, 3E,F, 4E,F**).

## Discussion

Approximately 40–60% of the Asians are characterized by a single eyelid or an absent superior palpebral fold (16). However, the double eyelid shape makes the eye look slightly larger and leads to an appearance of youthfulness and alertness. Therefore, upper eyelid blepharoplasty is gaining increasing popularity in the Far East. However, as the number of patients undergoing upper blepharoplasty is increasing rapidly and the expectations and demands on postoperative appearance have elevated significantly, there is a rise in the number of complaints of unsatisfactory postoperative double eyelid morphology. Presently, the correction of the most unsatisfactory double eyelid is mainly by surgery; however, a second surgery could aggravate the adhesion; also, the recovery phase was prolonged. Therefore, an alternative solution for unsatisfactory postoperative double eyelid is an urgent requirement.

The unsatisfactory postoperative double eyelid could be primarily attributed to tissue edema and/or scar formation. Corticosteroid injection and botulinum toxin injection have been used for scar treatment for a long time (13). Compared to botulinum toxin, corticosteroid injection is more economical and practical. Triamcinolone preparations produced greater particle aggregates (>500 μm), which were not present in betamethasone preparations. Eyelid skin is very thin, and triamcinolone would form deposits under the eyelid skin and is more difficult to absorb (17). In addition, betamethasone injection used in this study is composed of betamethasone dipropionate, and is absorbed slowly and metabolized gradually. The improvement in skin conditions is usually observed within 2–3 days after injection and the effects would last for 1–2 weeks and betamethasone is widely used for treatment of scar (13). Therefore, this retrospective study was designed to observe the curative effect of early intralesional betamethasone injection for the treatment of unsatisfactory postoperative double eyelid in 30 patients and evaluate the morphological changes subsequently.

The upper eyelid blepharoplasty causes tissue trauma, which leads to an inflammatory response and results in postoperative periorbital edema with discoloration. These features could mask the aesthetic outcomes and annoy the patient as well as the surgeon (10). Due to the varied circumstances of each person, some patients might suffer from severe eyelid edema for a prolonged period. However, persistent edema would lead to non-reversible morphological changes due to the biomechanical pulling and dilated skin and eventually to the formation of unsatisfactory postoperative double eyelid. In addition, severe postoperative edema and scar would affect the social status of young patients and even lead to some psychological problems. These psychological issues would influence the surgical satisfaction and further lead to contradiction between doctors and patients. Therefore, early intervention by intralesional corticosteroid injection could accelerate the recovery process and increase the patients' satisfaction. To minimize the postoperative edema and recovery time, one of the methods is to administer steroids, which are commonly utilized in most of the plastic and maxillofacial surgical procedures (18–20). Glucocorticoid inhibits the initial inflammation processes that include edema formation, fibrin deposition, capillary dilatation, migration of lymphocytes, and increased phagocytic activity. These manifestations decrease the permeability of vessels, thereby decreasing the degree of exudation and edema (21).

Multiple folds and sunken eyelids mainly occur due to the adhesion and then the formation of scar (8). Scar is an undesirable yet normal outcome of wound healing that develops in the final stage of wound healing (22, 23). The injury to the skin activates a physiological response that is categorized into four phases: coagulation, inflammation, proliferation, and remodeling (24). During the proliferation phase, the formation of collagen III and the creation of an extracellular matrix are induced by platelet-derived growth factor (PDGF) and transforming growth factor-beta (TGF-β), released by macrophage-activated fibroblasts (24, 25). Steroids mediate the effects of these growth factors by reducing the synthesis of collagen, altering extracellular matrix components, such as glycosaminoglycans, and reducing the level of proinflammatory mediators (26). The decrease in collagen synthesis after intralesional corticosteroid injection is speculated to be due to fibroblast hypoactivity, reduction in fibroblast density, or modification of the maturation of fibroblasts (26). Therefore, corticosteroid injection inhibits scar formation and could be used in the management of unsatisfactory double eyelid.

In this study, intralesional corticosteroid injection for the correction of unsatisfactory postoperative double eyelid achieved remarkable cosmetic improvement in most of the patients, and was also economical and practical. Although the corticosteroid injection might be associated with a number of adverse events (27), no side effects were detected in this study. Therefore, intralesional corticosteroid injection for the correction of the postoperative unsatisfactory double eyelid is safe and convenient, and provides a novel method for early intervention. Nevertheless, the present study has some disadvantages, such as small sample size and lack of comparison. In order to clarify the curative effect of corticosteroid injection on the treatment of unsatisfactory postoperative double eyelid, a prospective comparative study should be carried out in the future.

## Data Availability Statement

The raw data supporting the conclusions of this article will be made available by the authors, without undue reservation.

## Ethics Statement

The studies involving human participants were reviewed and approved by the ethics committee of Shanghai Ninth People's Hospital. Written informed consent for participation was not required for this study in accordance with the national legislation and the institutional requirements. Written informed consent was obtained from the individual(s) for the publication of any potentially identifiable images or data included in this article.

## Author Contributions

SZ and YZ were responsible for drafting the manuscript, analysis, and interpretation of the data. FY, DY, YY, and MZ contributed to the acquisition and analysis of data. YL and YF were involved in the design of the work, analysis of data, and approval of the final version to be published. All authors read and approved the final manuscript.

## Conflict of Interest

The authors declare that the research was conducted in the absence of any commercial or financial relationships that could be construed as a potential conflict of interest.

## Supplementary Material

Supplementary Video 1 The process of injection.
